# Chemokine C-C Motif Ligand 4 Gene Polymorphisms Associated with Susceptibility to Rheumatoid Arthritis

**DOI:** 10.1155/2018/9181647

**Published:** 2018-05-31

**Authors:** Shu-Jui Kuo, Chien-Chung Huang, Chun-Hao Tsai, Horng-Chaung Hsu, Chen-Ming Su, Chih-Hsin Tang

**Affiliations:** ^1^Graduate Institute of Clinical Medical Science, China Medical University, Taichung, Taiwan; ^2^Department of Orthopedic Surgery, China Medical University Hospital, Taichung, Taiwan; ^3^Division of Immunology and Rheumatology, Department of Internal Medicine, China Medical University Hospital, Taichung, Taiwan; ^4^School of Medicine, China Medical University, Taichung, Taiwan; ^5^Department of Biomedical Sciences Laboratory, Affiliated Dongyang Hospital of Wenzhou Medical University, Dongyang, Zhejiang, China; ^6^Chinese Medicine Research Center, China Medical University, Taichung, Taiwan; ^7^Department of Biotechnology, College of Health Science, Asia University, Taichung, Taiwan

## Abstract

Chemokine C-C motif ligand 4 (*CCL4*) gene is a chemokine-encoding gene, and the polymorphism of* CCL4* gene has been shown to predict risk of various diseases. We want to investigate whether the single nucleotide polymorphisms (SNPs) of the* CCL4* gene can predict the risk of rheumatoid arthritis (RA). Between 2007 and 2015, we recruited 217 patients diagnosed with RA and 371 control participants. Comparative genotyping of the rs1634507, rs10491121, and rs1719153 SNPs was performed. When compared with participants with the A/A genotype of rs1719153, those with the A/T genotype were less likely to develop RA, as were those with the A/T+T/T genotype. The protective effect of the T-containing genotype was even more prominent among females. Those with A/T in rs1719153 were 56% less likely to develop RA compared with females with A/A; a similar protective effect was seen for females with the A/T+T/T genotype compared with those with A/A. The GTEx database revealed that patients carrying the T/T genotype had lower levels of CCL4 gene expression than those carrying the A/A genotype. These results indicate that the nucleotide T over the rs1719153 is associated with decreased* CCL4* gene expression and decreased risk for RA.

## 1. Introduction

Rheumatoid arthritis (RA) is an autoimmune disease characterized by marked hypertrophy and hypervascularity of the synovial tissues and joint destruction, affecting around 1% of the global population [1, 2]. Despite the emergence of promising novel therapies in recent years that have enabled a substantial amount of RA patients to achieve disease remission with minimal or no symptoms, a substantial proportion of patients remain treatment-refractory and experience progressive joint and functional destruction or even premature mortality [3, 4]. The mortality rates among the RA patients are 1.5 ~ 1.6 times higher than those among the general population [5, 6]. The risks of major morbidities, including infection and pulmonary and renal disease, are also higher among the RA patients than among the general population [5]. The recognition that genetic factors account for up to 60% of the overall susceptibility and development of RA highlights the importance of research into genetic aberrations of this disease [3, 7, 8]. Investigations into RA genetics may help to facilitate risk prediction for individual patients and tailor their treatment accordingly [3].

Single nucleotide polymorphisms (SNPs) denote the single nucleotide variations occurring at specific sites in the genome with appreciable frequency within the population. Genotyping SNPs of a population and comparing the distribution frequency of SNPs among subgroups (e.g., controls and patients) are frequently utilized to examine disease risk and prognosis, including RA [9–11]. For example, the polymorphisms of the chemokine C-C motif ligand 4 (CCL4) gene influence gene expression and protein function and predict risk and prognosis of various diseases, including hepatocellular carcinoma, oral cancer, and psoriasis [12–14].The* CCL4 *belongs to a cluster of genes located in the chromosomal region 17q11-q21. The CCL4 protein acts as the chemokine being secreted under mitogenic signals and antigens and attracting monocytes, dendritic cells, natural killer cells, and other effector cells into the site of inflamed or damaged tissue [15, 16]. On the other hand, the interplays between chemokines and chemokine receptors play a pivotal role in RA pathogenesis by mediating leukocyte trafficking to the inflamed synovium [17–19]. Despite the well-known impact of chemokines on RA pathogenesis and the recognition that SNPs of* CCL4* gene, a chemokine-encoding gene, play important roles in a variety of human diseases, little is known about the association between* CCL4* SNPs and the risk of RA. In this study, we have evaluated the predictive capacity of three* CCL4* SNPs as candidate biomarkers for susceptibility to RA.

## 2. Materials and Methods

### 2.1. Participants

Between 2007 and 2015, we recruited 217 patients (mean age: 54.95 ± 11.10 years) diagnosed with RA according to the 2010 American College of Rheumatology criteria at Dongyang People's Hospital, China. A cohort of 371 healthy participants (mean age: 42.18 ± 19.12 years) without a history of RA served as the control group. All participants attended this hospital and were from the same geographic region. This study was approved by the Ethics Committee of Dongyang People's Hospital and it had appropriate institutional review board approval (2015-YB002). Written informed consent was obtained from all participants.

### 2.2. Selection of* CCL4* Polymorphisms

The* CCL4* SNPs selected for this study were identified from multiallelic copy number variation (CNV) profiles encompassing the q12 region of chromosome 17 that includes* CCL4* genes. Nonsynonymous SNPs rs1634507, rs10491121 and rs1719153 were chosen using the National Center for Biotechnology Information (NCBI) SNP database of genetic variation.

### 2.3. Genomic DNA Extraction

Genomic DNA was extracted from peripheral blood leukocytes using the QIAamp DNA Blood Mini Kit (Qiagen, Inc., Valencia, CA, USA) and dissolved in TE buffer (10 mM Tris, 1 mM EDTA; pH 7.8), quantified by OD_260_, and then stored at –20°C for further analysis.

### 2.4. Real-Time PCR

Sequencing of allelic discrimination for the* CCL4* SNP was assessed by the ABI StepOne™ real-time polymerase chain reaction (PCR) system (Applied Biosystems, Foster City, CA, USA) and analyzed using Software Design Specification version 3.0 software (Applied Biosystems) using the TaqMan assay. The primers and probes used in the analysis of the* CCL4* gene polymorphisms were rs1634507 (product ID: C_7451708_10), rs10491121 (product ID: C_11626804_10), and rs1719153 (product ID: C_12120537_10). The PCRs were performed in a total volume of 10 *μ*L containing 5 *μ*L of Master Mix, 0.25 *μ*L of probes, and 10 ng of genomic DNA. The real-time PCR reaction included an initial denaturation step at 95°C for 10 minutes, followed by 40 amplification cycles of 95°C for 15 seconds and 60°C for 1 minute [20, 21].

### 2.5. Bioinformatic Analyses

The study used the Genotype-Tissue Expression (GTEx) dataset (https://www.gtexportal.org/home/) to identify correlations between SNPs and* CCL4* expression levels. We hypothesized that this investigation into expression quantitative trait loci (eQTLs) would elucidate the functional role of phenotype-associated SNPs in RA disease processes.

### 2.6. Statistical Analysis

Between-groups differences were considered significant if *p* values were less than 0.05. The Chi-square analysis tested whether the SNP genotype distributions were in Hardy-Weinberg equilibrium. The Mann–Whitney U test and Fisher's exact test were utilized for between-groups demographic comparisons. Multiple logistic regression models adjusted for confounding covariates estimated the adjusted odds ratios (AORs) and 95% confidence intervals (CIs) for associations between genotype frequencies and the risk of RA or clinicopathological characteristics. All data were analyzed with the software program Statistical Analytic System version 9.1.

## 3. Results

All study participants were identified as Chinese Han ethnicity (Table 1). Compared to the RA cohort, the control group had a significantly higher proportion of younger-age participants (80.1% versus 67.7%;* p*=0.001) and fewer females (56.3% versus 83.4%;* p*< 0.001). At the time of blood sampling, 39.6% of the RA cohort were receiving tumor necrosis factor-alpha (TNF-*α*) inhibitors, 56.2% were receiving methotrexate, and 54.8% were receiving prednisolone. The majority (84.8%) of RA patients were rheumatoid factor- (RF-) positive.

Polymorphism frequencies in patients and controls are shown in Table 2. All genotypes were in Hardy-Weinberg equilibrium (p>0.05). The most frequent genotypes for SNPs rs10491121, rs1719153, and rs1634507 were A/G, A/T, and A/C, respectively. Compared with participants with the homozygous A/A genotype of SNP rs1719153, those with the heterozygous A/T genotype were significantly less likely to develop RA (AOR 0.67; 95% CI, 0.46 to 0.99; p<0.05), as were those with the A/T+T/T genotype (AOR 0.69; 95% CI, 0.48 to 0.99; p<0.05).

The protective effect of the T-containing genotype was even more prominent among females. Those with A/T in SNP rs1719153 were 56% less likely (AOR 0.56; 95% CI: 0.36-0.88) to develop RA compared with females with A/A; a similar protective effect was seen for females with the A/T+T/T genotype compared with those with A/A (AOR 0.57; 95% CI, 0.38 to 0.87) (Table 4). These findings suggested a protective effect of* CCL4* gene polymorphisms for RA, and the effects were more prominent among females.

An analysis of SNP rs1719153 evaluated the impact of the genotype on medication (TNF-*α* inhibitors, methotrexate, and prednisolone), RF clinical status, and ESR values (Table 3). No apparent associations were found between this genotype and medication use, clinical status, or serum inflammatory markers. No correlations between SNP and treatment regimen or serum pathological markers were observed among the females (data not shown).

**Figure 1:**
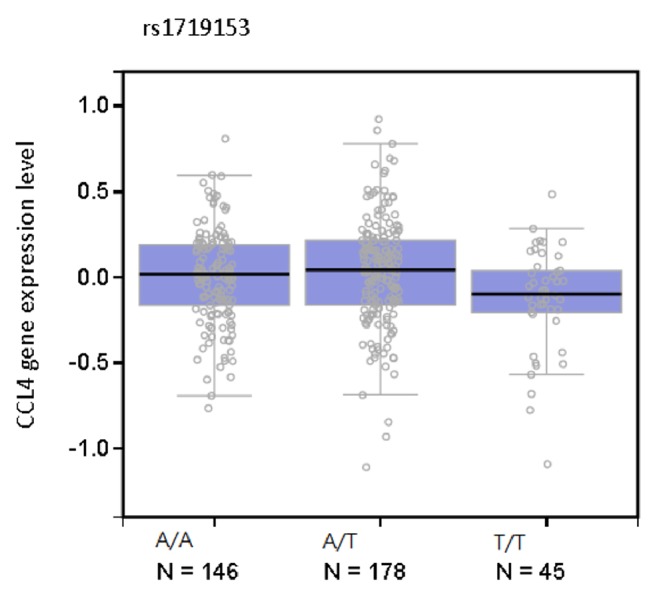
The GTEx dataset revealed a significant eQTL association between the rs1719153 genotype and expression of the* CCL4* gene in peripheral whole blood cells.

The GTEx database revealed that patients carrying the T/T genotype had lower levels of* CCL4* gene expression as compared with those carrying the wild-type A/A genotype (*p*< 0.001) (Figure 1). These results indicate that the nucleotide T over the rs1719153 is associated with the trend towards the decreased CCL4 gene expression.

## 4. Discussion

It is well recognized that susceptibility for RA disease is influenced by genetic and environmental factors [22, 23]. The recent development of biological-based antirheumatic therapies that target inflammatory pathways in RA has enabled increasing numbers of patients to achieve very low levels of disease activity, yet a substantial proportion of RA patients remain treatment-refractory [3, 24]. This issue underlines the importance of continuing to investigate the pathogenesis of RA. Genetic studies indicate that SNPs in particular susceptibility regions are associated with the risk of RA [3, 25]. Other studies suggest that searching for RA-related SNPs is a promising method to understand the pathogenesis of RA and for risk stratification [26, 27].

Macrophage Inflammatory Proteins (MIPs) belong to the family of chemotactic cytokines. In humans, there are two major forms, MIP-1*α* and MIP-1*β* that are now officially named CCL3 and CCL4, respectively. MIP proteins orchestrate acute and chronic inflammatory responses by recruiting proinflammatory cells and are thus a crucial component in the pathogenesis of asthma, granuloma formation, wound healing, arthritis, multiple sclerosis, pneumonia, and psoriasis [16]. Both CCL3 and CCL4 have been reported to be involved in the pathogenesis of RA. A genomewide haplotype association study showed that CCL3 was associated with RA susceptibility [28]. In an analysis of cartilage specimens from RA patients and multiorgan donors who served as controls, RT-PCR and flow cytometry revealed higher intracellular CCL4 expression levels among RA patients [29]. Another study evaluated site-specific levels of inflammatory mediators and corresponding Doppler ultrasound and MRI parameters using tissue samples from 58 synovial sites obtained from 25 patients with RA [30]. The study researchers showed that local CCL4 levels were associated with color Doppler ultrasound activity and RAMRIS synovitis scores. Other researchers have also noticed higher CCL4 expression levels in T cells from RA patients [31]. Histological examination of rheumatoid joints revealed extensive CCL4 expression in sites of lymphocyte infiltration and cell proliferation, leading the study researchers to conclude that CCL4 may substantially mediate the trafficking of reactive molecules involved in RA inflammatory processes. Despite evidence inferring a role for CCL4 in the pathogenesis of RA and the involvement of* CCL4* gene SNPs in various human diseases, few studies have investigated the relationship between CCL4 SNPs and risk of developing RA [12, 13, 32].

We have also begun to unravel the complicated pathogenesis of RA [2]. In this study, we sought to determine the prognostic accuracy of CCL4 SNPs in predicting RA onset. To the best of our knowledge, our study is the first to identify that the distribution of rs10491121, rs1719153, and rs1634507 SNPs is associated with RA pathogenesis. We also investigated the association of these* CCL4* SNPs with RA treatment regimens and serum pathological markers. We examined three* CCL4* SNPs among 371 controls and 271 RA patients. We found that in the general population the A/T genotype at SNP rs1719153 confers a lower risk for RA than the A/A genotype (AOR 0.67; 95% CI, 0.46 to 0.99). Female gender is a well-known risk factor for RA, so we performed a subanalysis of female participants. We demonstrated that the protective effect of the T-containing genotype is even more marked among females; those with the A/T genotype were half as likely to develop RA compared with females carrying the wild-type A/A genotype (AOR 0.56; 95% CI, 0.36 to 0.88). These findings have not been reported up to now.

Polymorphisms in the 3' flank region of a gene can influence gene expression [33]. We therefore examined whether the rs1719153 SNP identified to be protective against RA was associated with altered levels of* CCL4* gene expression. An examination of the GTEx database suggested that the T variant in the rs1719173 SNP is associated with a trend towards a lower level of* CCL4* expression as compared with the wild-type A nucleotide, reinforcing our findings. However, the rs1719173 SNP did not correlate with RF or ESR serum levels in the general population and separate analysis of female participants. The role of the rs1719173 SNP in the pathogenesis of RA deserves further investigation.

CCL4-like (CCL4L), a nonallelic copy of chemokine CCL4, differs from CCL4 by only one amino acid [34]. CCL4L has been shown to promote immune cell infiltration, including T helper type 1 cells, regulatory T cells, monocytes, and dendritic cells [32]. Two main types of CCL4L exist: the originally described variant, CCL4L1, and a second variant with a nucleotide change in the intron 2 region, CCL4L2 [35]. CCL4L gene copy number variation also modifies susceptibility to or control of HIV-1 infection [36, 37]. The copy number of CCL4L on the pathogenesis of RA warrants further investigation.

A major limitation to our study is that the observations of our study might be mere cross-relationship instead of actual causality. This is a ubiquitous limitation for similar studies and might be partially overcome by the deeper evaluation trying to select and analyze the relationships between all the known SNP elements.

## 5. Conclusions

Our study offers novel insights into* CCL4* SNPs in regard to RA susceptibility. The A/T genotype in the rs1719153 SNP was associated with decreased RA risk. This is the first study to demonstrate that a correlation exists between CCL4 polymorphisms and RA risk. CCL4 may prove to be a diagnostic marker and therapeutic target for RA therapy. Therapeutic agents that directly or indirectly modulate the expression of CCL4 may be the promising modalities for the treatment of RA.

## Figures and Tables

**Table 1:** Demographic profiles of the study participants.

Variable	Controls (N=371)	RA patients (N=217)	p value
Age			
≦60 years	297 (80.1%)	147 (67.7%)	0.001
>60 years	74 (19.9%)	70 (32.3%)	
Gender			
Female	209 (56.3%)	181(83.4%)	<0.001
Male	162 (43.7%)	36 (16.6%)	
RF-positive		33 (15.2%)	
ESR			
<20 mm/h		108 (49.8%)	
≧20 mm/h		109 (50.2%)	
TNF inhibitor			
NO		131 (60.4%)	
YES		86 (39.6%)	
Methotrexate			
NO		95 (43.8%)	
YES		122 (56.2%)	
Prednisolone			
NO		98 (45.2%)	
YES		119 (54.8%)	

RA: rheumatoid arthritis; RF: rheumatoid factor; ESR: erythrocyte sedimentation rate; TNF: tumor necrosis factor.

**Table 2:** Polymorphism frequencies among the study participants.

SNPs	Controls	RA patients	OR (95% CI)	AOR (95% CI)
	(N=371)	(N=217)		
rs10491121				
A/A	94 (25.3%)	54 (24.9%)	1.00	1.00
A/G	175 (47.2%)	105 (48.4%)	1.04 (0.69-1.58)	0.82 (0.53-1.28)
G/G	102 (27.5%)	58 (26.7%)	0.99 (0.62-1.58)	0.91 (0.56-1.49)
A/G+G/G	277 (77.7%)	163 (75.1%)	1.02 (0.70-1.51)	0.85 (0.56-1.27)
rs1719153				
A/A	162 (43.7%)	80 (36.9%)	1.00	1.00
A/T	164 (44.2%)	108 (49.8%)	1.33 (0.93-1.91)	0.67 (0.46-0.99)^#^
T/T	45 (12.1%)	29 (13.4%)	1.31 (0.76-2.24)	0.77 (0.44-1.35)
A/T+T/T	209 (56.3%)	137 (63.2%)	1.33 (0.94-1.87)	0.69 (0.48-0.99)^#^
rs1634507				
C/C	161 (43.4%)	90 (41.5%)	1.00	1.00
A/C	165 (44.5%)	98 (45.2%)	1.06 (0.74-1.52)	0.81 (0.55-1.19)
A/A	45 (12.1%)	29 (13.4%)	1.15 (0.68-1.97)	0.89 (0.51-1.57)
A/C+A/A	210 (56.6%)	127 (58.6%)	1.08 (0.77-1.52)	0.83 (0.58-1.19)

Data are represented as N (%).

^#^ *p*< 0.05.

SNP: single nucleotide polymorphism; RA: rheumatoid arthritis; OR: odds ratio; CI: confidence interval; AOR: adjusted odds ratio.

**Table 3:** Frequencies of CCL4 rs1719153 polymorphisms among RA patients, stratified by medications, RF, and ESR status.

Variables (general population)		A/A	A/T + T/T	AOR (95% CI)
TNF-*α* inhibitor	No	48	83	1
	Yes	32	54	0.98 (0.56-1.71)
Methotrexate	No	38	57	1
	Yes	43	80	1.27 (0.73-2.21)
Prednisolone	No	33	65	1
	Yes	47	72	0.78 (0.45-1.36)
RF	Negative	13	20	1
	Positive	67	117	1.14 (0.53-2.43)
ESR (mm/h)	< 20	36	72	1
	> 20	44	65	0.74 (0.43-1.29)

RA: rheumatoid arthritis; RF: rheumatoid factor; ESR: erythrocyte sedimentation rate; CI: confidence interval; AOR: adjusted odds ratio; TNF-*α*: tumor necrosis factor-alpha.

**Table 4:** Polymorphism frequencies among the female participants.

Variable	Control females	RA females	OR (95% CI)	AOR (95% CI)
	(N=209)	(N=181)		
rs10491121				
A/A	64 (30.6%)	44 (24.3%)	1.00	1.00
A/G	92 (44.0%)	88 (48.6%)	1.39 (0.86-2.25)	0.70 (0.43-1.14)
G/G	53 (25.4%)	49 (27.1%)	1.35 (0.78-2.32)	0.76 (0.44-1.32)
A/G+G/G	145 (69.4%)	137 (72.3%)	1.37 (0.88-2.15)	0.72 (0.45-1.14)
rs1719153				
A/A	101 (48.3%)	64 (35.4%)	1.00	1.00
A/T	83 (39.7%)	90 (49.7%)	1.71 (1.11-2.64)^#^	0.56 (0.36-0.88)^#^
T/T	25 (12.0%)	27 (14.9%)	1.70 (0.91-3.19)	0.60 (0.31-1.13)
A/T+T/T	108 (51.7%)	117 (64.6%)	1.71 (1.14-2.57)^#^	0.57 (0.38-0.87)^##^
rs1634507				
C/C	101 (48.3%)	75 (41.4%)	1.00	1.00
A/C	81 (38.8%)	81 (44.8%)	1.35 (0.88-2.07)	0.72 (0.46-1.11)
A/A	27 (12.9%)	25 (13.8%)	1.25 (0.67-2.32)	0.86 (0.46-1.63)
A/C+A/A	108 (51.7%)	106 (58.6%)	1.32 (0.89-1.97)	0.75 (0.50-1.13)

^#^p < 0.05; ^##^p < 0.01.

RA: rheumatoid arthritis; OR: odds ratio; CI: confidence interval; AOR: adjusted odds ratio.

## Data Availability

The original data of this study can be offered by the corresponding authors on reasonable request under the consent of the IRB board of the Dongyang People's Hospital.
